# Experimental Mutations in Superoxide Dismutase 1 Provide Insight into Potential Mechanisms Involved in Aberrant Aggregation in Familial Amyotrophic Lateral Sclerosis

**DOI:** 10.1534/g3.118.200787

**Published:** 2019-01-08

**Authors:** Anthony M. Crown, Brittany L. Roberts, Keith Crosby, Hilda Brown, Jacob I. Ayers, P. John Hart, David R. Borchelt

**Affiliations:** * Department of Neuroscience, Center for Translational Research in Neurodegenerative Disease, McKnight Brain Institute, University of Florida, Gainesville, FL 32610; † College of Arts and Sciences, McKnight Brain Institute, University of Florida, Gainesville, FL 32610; ** SantaFe HealthCare Alzheimer’s Disease Research Center, McKnight Brain Institute, University of Florida, Gainesville, FL 32610; ‡ Department of Veterans Affairs, South Texas Veterans Health Care System, San Antonio, TX 78229; § Department of Biochemistry University of Texas Health Science Center, San Antonio, TX 78229

**Keywords:** mutagenesis, structure, protein aggregation, cell models

## Abstract

Mutations in more than 80 different positions in superoxide dismutase 1 (SOD1) have been associated with amyotrophic lateral sclerosis (fALS). There is substantial evidence that a common consequence of these mutations is to induce the protein to misfold and aggregate. How these mutations perturb native structure to heighten the propensity to misfold and aggregate is unclear. In the present study, we have mutagenized Glu residues at positions 40 and 133 that are involved in stabilizing the β-barrel structure of the native protein and a critical Zn binding domain, respectively, to examine how specific mutations may cause SOD1 misfolding and aggregation. Mutations associated with ALS as well as experimental mutations were introduced into these positions. We used an assay in which mutant SOD1 was fused to yellow fluorescent protein (SOD1:YFP) to visualize the formation of cytosolic inclusions by mutant SOD1. We then used existing structural data on SOD1, to predict how different mutations might alter local 3D conformation. Our findings reveal an association between mutant SOD1 aggregation and amino acid substitutions that are predicted to introduce steric strain, sometimes subtly, in the 3D conformation of the peptide backbone.

Approximately 10–20% of ALS cases with a family history have mutations in Cu-Zn superoxide dismutase (SOD1) (Rosen *et al.* 1993). The native form of this ubiquitously expressed antioxidant enzyme is a homodimer of two 153 amino acid subunits (McCord and Fridovich 1969; Fridovich 1974). The native structure of the protein contains eight β-strands, a catalytic copper ion, a structurally important zinc ion, an electrostatic loop element that forms a portion of the active site funnel, and an intramolecular disulfide bond between cysteine 57 and cysteine 146 (Parge *et al.* 1992; Ogihara *et al.* 1996; Hart *et al.* 1999). The vast majority of the more than 160 SOD1 mutations associated with ALS are missense point mutations {http://alsod.iop.kcl.ac.uk}. Some of these point mutations have been shown to diminish normal enzyme activity or accelerate protein turnover; whereas other mutations have limited effects on activity or protein half-life (Gurney *et al.* 1994; Borchelt *et al.* 1994; Nishida *et al.* 1994; Wong *et al.* 1995; Wiedau-Pazos *et al.* 1996; Ratovitski *et al.* 1999; Hayward *et al.* 2002; Jonsson *et al.* 2004; Wang *et al.* 2005; Jonsson *et al.* 2006b). In autopsy studies of SOD1 ALS cases, SOD1 immuno-reactive inclusions in spinal cord are commonly, but not uniformly, found pathologic features (Shibata *et al.* 1996a, 1996b; Shaw *et al.* 1997; Sasaki *et al.* 1998; Kokubo *et al.* 1999; Kato *et al.* 2001; Takehisa *et al.* 2001; Tan *et al.* 2004; Ohi *et al.* 2004; Jonsson *et al.* 2008; Suzuki *et al.* 2008; Kerman *et al.* 2010; Hineno *et al.* 2012; Sábado *et al.* 2013; Nakamura *et al.* 2014; Steinacker *et al.* 2014). Studies in a variety of model systems have reported that the ALS-associated mutations in SOD1 cause the protein to be more prone to misfold and aggregate (Johnston *et al.* 2000; Shinder *et al.* 2001; Wang *et al.* 2002; Elam *et al.* 2003; Jonsson *et al.* 2004; Prudencio *et al.* 2009). Misfolded SOD1 has also been described as a pathologic feature of sporadic ALS using antibodies that are preferentially reactive to non-natively folded SOD1 (Bosco *et al.* 2010; Forsberg *et al.* 2010; Grad *et al.* 2014). Other studies, however, have disputed these findings (Brotherton *et al.* 2012; Ayers *et al.* 2014b; Da Cruz *et al.* 2017).

The majority of studies in cell and mouse models have suggested that mutant SOD1 is generally more prone to misfold and aggregate, but the distinction is not absolute. *In vitro*, purified WT SOD1 can be readily induced to aggregate into amyloid-like fibrillary structures by de-metalation (Cu) and disulfide reduction (Rakhit *et al.* 2002; DiDonato *et al.* 2003; Furukawa and O’Halloran 2005; Chattopadhyay *et al.* 2008, 2015; Banci *et al.* 2008; Furukawa *et al.* 2010; Pratt *et al.* 2014; Abdolvahabi *et al.* 2015, 2016). Similarly, de-metalation and reduction of purified fALS mutant SOD1 also induces fibrillary amyloid structures (Münch and Bertolotti 2010; Vassall *et al.* 2011; Pratt *et al.* 2014; Abdolvahabi *et al.* 2016). In these *in vitro* assays, the differences in aggregation propensity between WT and fALS mutant SOD1 are variably evident (Banci *et al.* 2008; Furukawa *et al.* 2010). Some studies have reported that fALS mutants aggregate more rapidly *in vitro* (Münch and Bertolotti 2010; Vassall *et al.* 2011; Pratt *et al.* 2014), and are more prone to oligomerization (Furukawa and O’Halloran 2005; Münch and Bertolotti 2010; Vassall *et al.* 2011). However, a recent study that examined the rates of aggregation for a panel of ALS mutants *in vitro*, using Thioflavin T assays, reported that the assay exhibits considerable variability making it difficult to reliably say whether purified SOD1 with fALS mutations reproducibly aggregates faster (Abdolvahabi *et al.* 2016). Additionally, competition between the formation of amyloid-like structures that bind Thioflavin T, the reporter dye used for assessing SOD1 aggregation, and the formation of amorphous aggregates that do not bind the dye is a confounding factor in assessing SOD1 aggregation (Abdolvahabi *et al.* 2016). Interestingly, SOD1 aggregates generated *in vitro* in the presence of lipids tend to be more amorphous in structure (Choi *et al.* 2011). Purified Holo pseudo WT-SOD1 (pSOD1, fully metallated, presumably with a correct intramolecular disulfide bond, encoding mutations C6A, C111S) is slow to aggregate *in vitro* (days as compared to hours for demetalled SOD1) and generally forms non-amyloid aggregates (Hwang *et al.* 2010). The addition of fALS mutations, such as A4V, A4T, G85R, G93A, and I149T, to pseudo WT-SOD1 generally appears to decrease the lag phase for aggregate formation, but this effect is not consistent across all mutants; A4S, G93D, G93V, and G93S show little or no difference from Holo pSOD1 (Hwang *et al.* 2010). Collectively, these studies demonstrate that SOD1 could be viewed as inherently prone to aggregation, and illustrate some of the challenges in defining whether disease-associated mutations alter aggregation propensity *in vitro*.

In mouse models of WT and mutant SOD1 over-expression, mutant SOD1 appears to be significantly more prone to aggregate (Graffmo *et al.* 2013). Mice expressing high levels of human WT SOD1 develop ALS-like paresis but at much later ages than mice expressing equivalent levels of mutant SOD1. At end-stage, the levels of mis-folded, aggregated SOD1 in paralyzed mice expressing WT SOD1 were about half the level in paralyzed mice expressing the G93A mutant SOD1 (Graffmo *et al.* 2013). Mice expressing WT-SOD1 fused to yellow fluorescent protein (SOD1:YFP) do not develop ALS-like paralysis and do not show evidence of WT-SOD1:YFP aggregation at advanced ages; whereas equivalently expressed fALS mutant G85R-SOD1:YFP produces paralysis with obvious inclusion pathology (Wang *et al.* 2009). Similar data for SOD1:YFP fusion proteins has been described in cultured cell models of over-expression (Corcoran *et al.* 2004; Matsumoto *et al.* 2005; Turner *et al.* 2005; Fei *et al.* 2006; Zhang and Zhu 2006; Urushitani *et al.* 2008; Witan *et al.* 2009). We have demonstrated that SOD1:YFP fusions with fALS mutations A4V, G37R, G85R, D101N, C111Y, S134N all readily form fluorescent inclusions when over-expressed in cultured cells (Prudencio and Borchelt 2011; Roberts *et al.* 2012). WT-SOD1:YFP displays a diffuse distribution in the cytoplasm and readily diffuses out of permeabilized cells whereas mutant SOD1 fused to YFP forms immobile inclusions (Prudencio and Borchelt 2011). Untagged versions of SOD1 with ALS mutations (>40 mutants tested) form detergent insoluble, sedimentable, aggregates when transiently over-expressed in cell models (Wang *et al.* 2003; Karch and Borchelt 2008; Prudencio *et al.* 2009). SOD1:YFP tagged variants of mutant SOD1 that form inclusions are similarly sedimentable and detergent-insoluble (Prudencio and Borchelt 2011). Collectively, this body of work demonstrates that fALS mutations in SOD1 share a common feature of promoting aggregation of the protein, and that visualizing aggregation by expressing YFP fusion proteins in cells models is a useful approach.

The objective of the current study was to investigate how amino acid substitutions may induce the aggregation of SOD1. As outlined above, there have been many studies of aggregation by SOD1 with fALS point mutations, but there has not been an assessment of how tolerant a given position might be for different types of substitutions. Here, we focus on two amino acid positions in SOD1 that are in critical structural locations in the protein, Glu 40 and Glu 133. Glu 40 is located in a loop domain that is juxtaposed to Lys 91 within a portion of the protein that forms an element called a β-barrel plug, which creates a barrier to exclude solvent from the hydrophobic core of SOD1’s beta barrel structure (Figure 1)(Hart *et al.* 1998, 1999). Glu 133 is located in an alpha helical segment in a loop element that is critical in binding Zn (Figure 1). These amino acids were mutated to encode either ALS mutations or specific experimental mutations within SOD1:YFP fusion constructs, and we then assessed aggregation of the protein. Our experimental data suggests that mutations that disrupt van der Wäals interactions or introduce steric strain are poorly tolerated and are associated with a higher propensity to aggregate.

**Figure 1:**
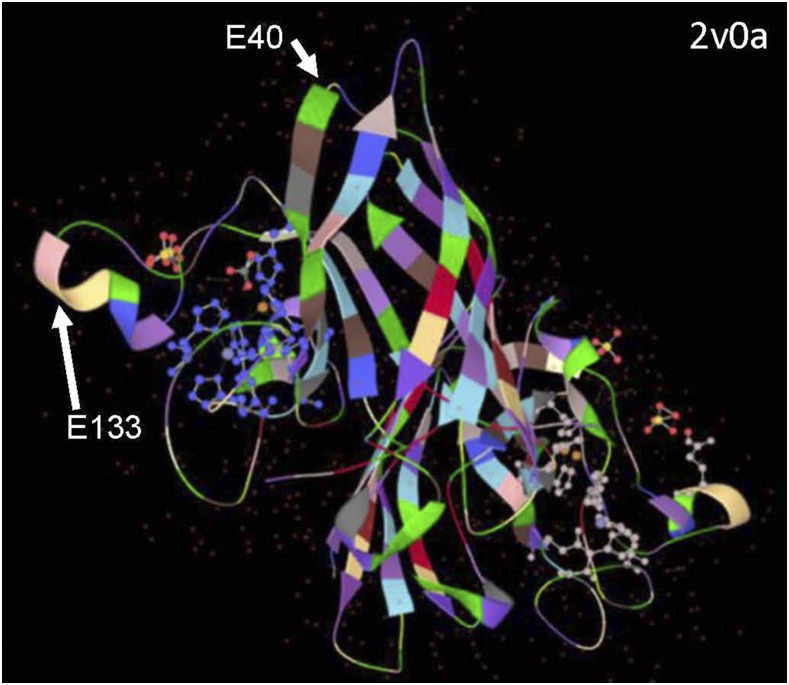
Image of homodimeric SOD1 structure. A representation of SOD1 homodimer structure (protein data bank 2v0a) captured from a 3-D representation created in LiteMol through the Protein Data Bank in Europe (https://www.ebi.ac.uk/pdbe/entry/pdb/2V0A). The locations of E40 and E133 are noted in one of the two subunits visible in the image.

## Materials And Methods

### Generation of mutant SOD plasmids

Mutations were introduced in human *SOD1* cDNA using oligonucleotide primers encoding the desired mutation with QuikChange mutagenesis kits (Agilent Technologies, Santa Clara, CA). The cDNA gene for *SOD1* was mutagenized in pEF.BOS vectors (Mizushima and Nagata 1990) that encode WT-SOD1:YFP cDNA (Prudencio and Borchelt 2011) as the template. The PCR reaction used Platinum *Pfx* polymerase (Invitrogen/ThermoFisher, Waltham, MA) and 2X Pfx buffer concentration to accommodate the large plasmids that were amplified. The PCR reaction products were digested with Dpn1 to remove template and then transformed into NEB-10β competent cells (New England Biolabs, Ipswich, MA) following standard protocols. Large scale preparations of plasmid DNA for transfection were prepared by CsCl gradient purification. The SOD1:YFP coding elements of all plasmids produced from CsCl purification was verified by DNA sequence analysis.

### Transient Transfections

Expression plasmids were transiently transfected into Chinese hamster ovary (CHO) cells grown on 60-mm poly-D-lysine-coated dishes (1 plate for each DNA construct). Upon reaching 95% confluency, cells were transfected with Lipofectamine 2000 (Invitrogen/ThermoFisher). The cells were then incubated at 37° in a CO_2_ incubator for 24 hr at which time images of random fields of view at 20x and 40 × magnification were captured using an AMG EVOS_fl_ digital inverted microscope for fluorescence. The cells were returned to the incubator for 24 hr before images were captured again. The transient transfections were repeated at least 3 times for each construct. The images from multiple transfections were analyzed and cells showing YFP fluorescence and cells showing fluorescent inclusions were counted.

### Immunoblotting

At 48 hr post-transfection, CHO cells were washed from the plate in 1X PBS, and then centrifuged at 3000xg rpm for 5 min before resuspension in 1X PBS with protease inhibitor cocktail (Sigma, St. Louis, MO, USA). Cells were disrupted with a probe sonicator by three 10 sec bursts, and protein concentrations determined with BCA assay (Pierce/ThermoFisher, Waltham, MA). From these lysates, 5µg of protein was separated by electrophoresis in 18% TG-SDS PAGE gels (Invitrogen/ThermoFisher). After transfer to nitrocellulose membrane, the SOD1:YFP proteins were revealed by incubation with SOD1 rabbit polyclonal antibody (Ratovitski *et al.* 1999) and detection by enhanced chemiluminesence as previously described (Ayers *et al.* 2014a).

### Molecular Modeling

The observed conformations (rotamers) adopted by the side chains of residues E40, K91 and E133 were visualized by superimposing all of the wild type SOD1 subunits available in the protein data bank (PDB). The possible structural consequences of amino acid substitutions at positions E40 and E133 were examined *in silico* using the structure of wild type human SOD1 refined to 1.15 Å as the template [pdb code 2v0a (Strange *et al.* 2007)]. Substitutions for E40 and E133 were introduced into the human wild type template structure and all possible backbone dependent and independent rotamers for the substituted residues were scrutinized using the mutagenesis wizard in program PyMol (The PyMol Molecular Graphics System, Version 1.7.0, Schrödinger, LLC).

### Data and Reagent Availability

All recombinant DNA constructs used in this study are available upon request. The authors confirm that all data necessary for confirming the conclusions of the article are present within the article, figures, tables, and supplemental materials. Supplemental material available at Figshare: https://doi.org/10.25387/g3.7474946.

## Results And Discussion

**Figure 2:**
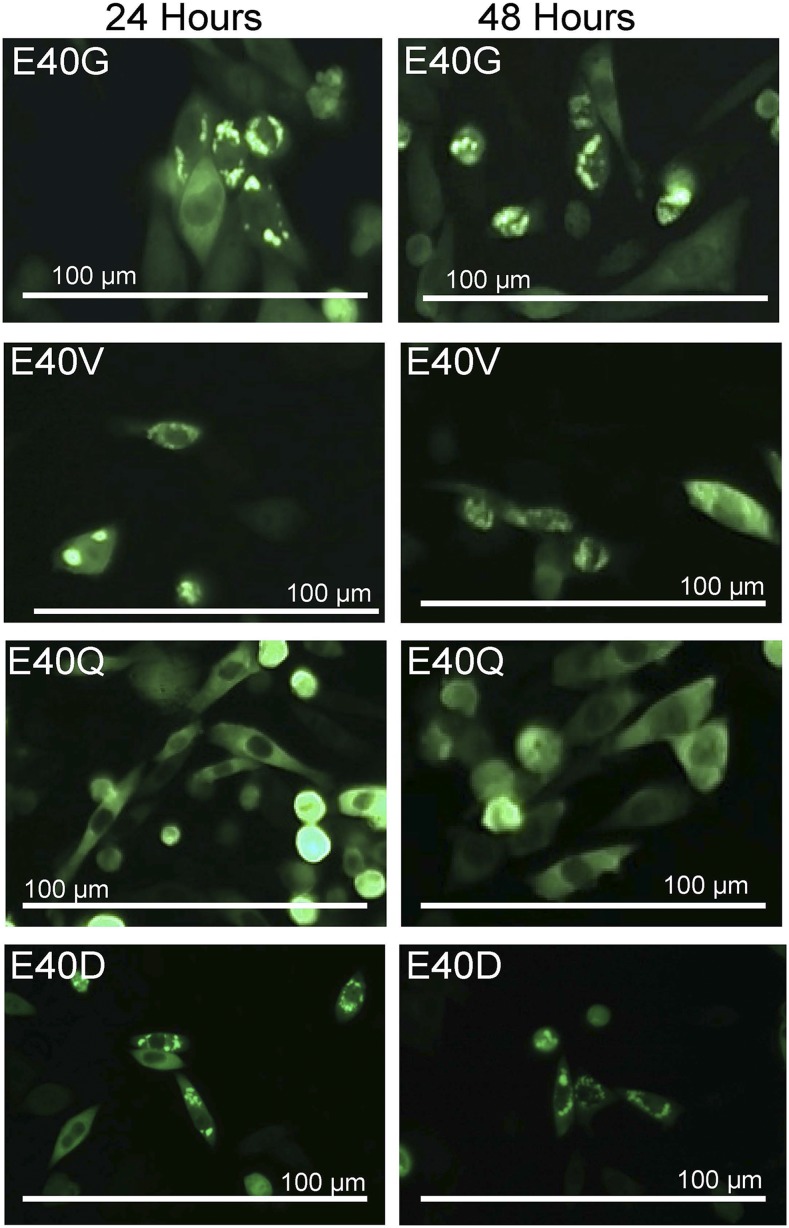
Example of the images that were scored to assess the frequency of inclusion formation by variants SOD1 encoding amino acid substitutions at residue E40. Recombinant DNA vectors encoding SOD1 fused in-frame to YFP were transfected into CHO cells as described in Methods and images were captured at 24 and 48 hr. The E40G mutation has been associated with ALS, whereas the E40V, E40Q, and E40D mutations are experimental substitutions.

In prior studies, we have used both HEK293 and Chinese Hamster Ovary (CHO) cells to visualize the aggregation of mutant SOD1 fused to YFP, finding similar results (Prudencio and Borchelt 2011; Roberts *et al.* 2012). In the present study, we used the CHO cell model because these cells have a large cytosol compartment that allows for a clear assessment of inclusion formation by the fusion proteins. Prior studies have established that the formation of inclusions by these fusion proteins in cell models correlates to changes in protein solubility and mobility (Prudencio and Borchelt 2011). Within the time frame of these 48 hr transfections, CHO cells do not show significant cell death due to mutant SOD1 expression (Prudencio and Borchelt 2011). Additionally, in this CHO model of transient transfection, the SOD1:YFP proteins are highly over-expressed in a subset of cells, overwhelming any modulation of aggregation by chaperone function that could occur and minimizing variabilities in protein half-life (Prudencio *et al.* 2009; Ayers *et al.* 2014a). In previous studies of SOD1 in these over-expression cell models, we have determined that both WT and mutant SOD1 are largely deficient in Cu ions and that mutant SOD1 is substantially less able to form the normal intramolecular disulfide bond associated with full maturation (Ayers *et al.* 2014a). Thus, the model assesses the inherent propensity of immature mutant SOD1 to aggregate. The use of the visual assay helps to control for variations in expression levels as fluorescence intensity can be used as a gauge for relative expression. As described in Methods, three transient transfections were performed for each construct and representative images were captured at 24 and 48 hr. For quantitative analysis of aggregate formation, cells expressing the fusion proteins were identified by fluorescence and scored for the presence of inclusion-like structures. Examples of what the observer scored are shown in Figure 2. Cells were scored as having inclusions when the observer could clearly discern the presence of multiple highly fluorescent puncta within the cytosol. A subset of cells in these images show changes in morphology in which they adopt a rounded appearance due to detachment from the culture surface. The rounded cells were generally not counted because they were slightly out of focus relative to the flatter cells.

**Figure 3:**
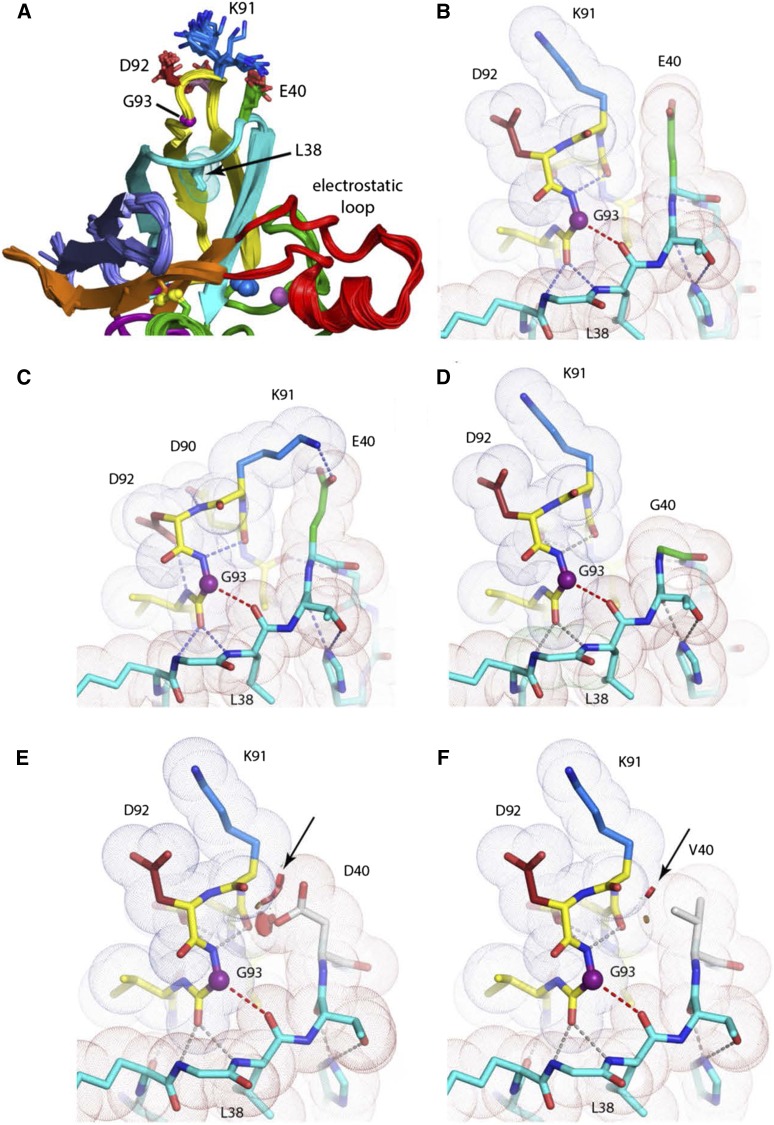
Local structure near β-barrel plug (L38) in WT SOD1 and predicted impact of E40 substitutions. *A*, Superposition of 36 unique protomers of WT hSOD1 available in the Protein Data Bank [see Table S2 in (Ayers *et al.* 2014b) ] reveals an ensemble of conformers of the side chains of E40, D90, K91, and D92. The color-coding of consecutive beta-strands includes residues in connecting loops. Strands 1-2 (blue), strands 3-4 (cyan), strands 5-6 (yellow), strands 7-8 (orange), electrostatic loop (loop VII, red). *B and C*, The packing of the 3-4 loop with the 5-6 loop maximizes van der Wäals contacts. The extended side chain of E40 makes favorable contacts with atoms of K91, sealing the apolar interior of the β-barrel from bulk solvent. *D*, The aggregating G40 variant loses multiple van der Wäals interactions relative to its E40 counterpart. *E*, The aggregating D40 variant preserves charge, but introduces steric clashes (red discs, arrow) with residues in the 5-6 loop (see additional D40 rotamers/clashes in Figure S1). *F*, The short, branched side chain of the aggregating V40 variant also makes unfavorable contacts (red discs, arrow) with residues of the 5-6 loop (see text).

The E40 and E133 positions that encode Glu were first mutated to amino acid substitutions that are known to be associated with ALS. Mutation of E40 to G is listed in the ALSOD database {http://alsod.iop.kcl.ac.uk} as a disease-causing mutation as are two different types of mutations at E133 (E133del, E133V), however, data on the number of ALS patients with these mutations is limited. The E40 position was also mutated experimentally to non-conservative Val or Gln residues, and a conservative Asp residue. As expected for mutations associated with fALS, the E40G variant produced inclusions at relatively high frequency (Figure 2; Table 1). Mutation of E40 to V also produced inclusions (Figure 3), but less frequently than the E40G-SOD1:YFP variant (Table 1). The E40Q SOD1:YFP construct did not form any obvious inclusions at 24 or 48 hr (Figure 2; Table 1; the image shown in Figure 2 has several examples of rounded out of focus cells which appear much brighter due to light diffraction). To discern the effect of conservative mutations at this site, we generated an E40D variant, finding that this variant produced inclusions at frequencies well above background at both 24 and 48 hr (Figure 2, Table 1).

**Table 1:** Summary of inclusion formation by mutant SOD1 constructs examined with predicted structural consequence caused by each mutation

SOD1 Variant	# cells counted (24 hr)	# cells w/ inclusions (24 hr)	% cells w/ inclusions (24 hr)	# cells counted (48 hr)	# cells w/ inclusions (48 hr)	% cells w/ inclusions (48 hr)	Predicted structural consequence
WT	656	5	≤ 1%	742	6	1%	N/A
G93A*	333	78	23%	503	243	48%	Steric clash
E40D	187	64	34%	267	61	23%	Loss of van der Wäals, Steric clash
E40G*	267	78	29%	238	186	78%	Loss of van der Wäals
E40Q	217	0	0%	229	0	0%	None
E40V	116	28	24%	147	37	25%	Steric clash
E133D	219	0	0%	197	0	0%	None
E133G	221	0	0%	288	0	0%	None
E133L	275	0	0%	373	0	0%	None
E133M	243	0	0%	265	0	0%	None
E133V*	324	38	12%	254	62	24%	Steric clash
E133Del*	192	12	6%	255	60	24%	Loss of H-bonds

* sequence variants associated with ALS.

To identify a potential molecular basis for our observations, we examined the available data on SOD1 3-D structure to identify features of these amino acid substitutions that could cause aggregation. To obtain an unbiased representation of the hSOD1 WT structure around E40, we examined 7 different WT structures from the protein data bank, including 32 protomers [same approach as described in (Ayers *et al.* 2014b)], and structurally aligned each protomer to generate a representative or “consensus” structure. K91 is positioned at the apex of the type I β-turn between strands 5 and 6, whereas E40 is located in the omega loop between β-strands 3 and 4. The type 1 β-turn and omega loop are in contact, coming together to close one end of the β-barrel (Figure 3A & B). At the midpoint of the omega loop, L38 acts as an apolar “plug” of the β-barrel where it engages in multiple apolar interactions with residues coming from neighboring structural elements (*e.g.*, V14, I35, F45, V87, A89, A95, V119, and L144, Figure 3A). Mutations associated with ALS have been found at all but two of these positions (35 and 119) {http://alsod.iop.kcl.ac.uk}. The side chain of K91 is observed in several conformations (Figure 3A). The β-hairpin and omega loop elements are highly complementary in shape and are associated primarily through van der Wäals contacts and several main-chain to main-chain hydrogen bonds (Figure 3B). The absence of side chains at G37, G41, and G93 (purple sphere, Figure 3B) is important for this shape complementarity. Mutations of G37 to R or V, G41 to S or D, and G93 to A, C, D, S, R, or V have been associated with ALS {http://alsod.iop.kcl.ac.uk}. K91 donates a hydrogen bond to the side chain of D92 in five protomers, makes no hydrogen bond in 23 protomers (Figure 3B), and donates a hydrogen bond to the side chain of E40 in 8 protomers (Figure 3C). The side chain of E40 is essentially invariant in all structures, engaging in van der Wäals interaction with the backbone of the loop formed by residues 90-93 (Figure 3B & C). Mutation of E40 to G is predicted to weaken van der Wäals contacts and diminish strength of interaction between the two loop domains (Figure 3D). Mutation of E40 to D is predicted to weaken van der Wäals contacts or introduce a clash, depending on the rotamer examined (Figure 3E, Figure S1), while mutation to V is predicted to introduce a steric clash (Figure 3F). Asp cannot adopt the same extended conformation as Glu (it is one carbon shorter and there is no rotamer that permits it). By contrast, Gln at position 40 is predicted to behave identically to Glu because its side chain is the same length and can access the same rotamer conformations. In addition, unlike Asp or Glu, a Gln side chain at position 40 could form a hydrogen bond with the carbonyl oxygen or side chain nitrogen of K91. When E40 is mutated to Val, however, there is no way to avoid a steric clash with residues of the β-hairpin as all possible rotamers of V40 introduce steric clashes that would disrupt the surface complementarity between the β-hairpin and omega loop elements in this region (Figure 3F). In 23 protomers, K91 makes no hydrogen bond (see Figure 3A), suggesting that the loss of favorable aliphatic interactions between the β and γ carbons of E40 with the backbone of the 93 loop and the β carbon of K91 is deleterious. Thus, we can explain the behavior of the mutations made at E40 based on preservation or abolition of structure-stabilizing van der Wäals interactions between residues E40 and K91, located in two juxtaposed β-hairpins structures.

To analyze how mutations in the Zn loop may produce aggregation, position E133 was mutated to ALS mutants E133V or an in-frame deletion (E133del), and experimental substitutions to Gly, Leu, Met, or to Asp (all as fusions of SOD1 with YFP). As expected for mutations associated with fALS, the E133V, and E133del variants produced inclusions at relatively high frequency (Table 1, Figure S2). Cells expressing the experimental constructs, E133G-SOD1:YFP, E133L-SOD1:YFP, E133M-SOD1:YFP, and E133D-SOD1:YFP, did not develop inclusion-like structures by 24 or 48 hr (Table 1; Figure S3). We verified similar levels of expression for each of these variants by immunoblotting (see Figure S4). Collectively, these findings indicated that position E40 has relatively low tolerance for mutation, even a conservative Glu to Asp mutation, whereas the E133 positon was more tolerant of mutation.

**Figure 4:**
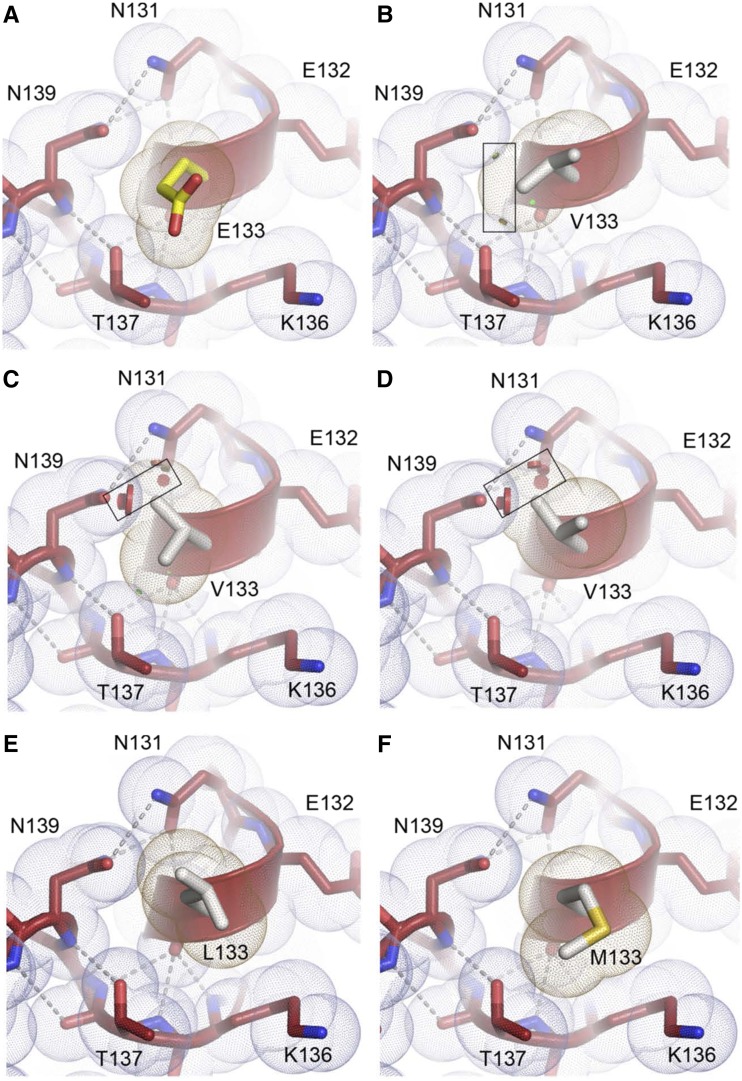
Local structure of the short alpha-helix in loop VII and predicted effects of E133 substitutions. *A*, The branched portion of E133 extends into the solvent far enough that there are no steric clashes with residues T137, N131, and N139. The reciprocal hydrogen bonds between N131 and N139 are critical to maintain the conformation of loop VII. *B-D*, The short, branched side chain of the aggregating V133 variant clashes with atoms of T137, N131, and N139 (discs, boxed) and these unfavorable interactions are predicted to disrupt the hydrogen bonds between N131 and N139. *E and F*, The extra methylene spacer in the non-aggregating L133 and M133 variants relative to the aggregating V133 variant relieves unfavorable steric clashes.

The Glu at residue 133 is positioned within an alpha helical segment of the protein in the Zn loop (loop IV). The two disease causing mutations, E133del and E133V, both induced aggregation of SOD1, but other nonconservative mutations including E133G, E133M, and E133L did not. The alpha helical segment that contains E133 is stabilized by an extensive network of hydrogen bonds and extensive van der Wäals interactions (Figure 4A). The loss of a residue at position 133 would be devastating to the structure of the electrostatic loop (loop VII) because residues C-terminal to 133 would become out-of-register, misaligned for stabilizing hydrogen bonds and optimal van der Wäals packing. As with E40V, the E133V mutation is likely to be destabilizing because the short, branched nature of the Val chain is predicted to clash with the nearby side chains Asn 127 and Asn 135; the reciprocal bonds between which are integral to the stabilization of the conformation of the electrostatic loop (Figure 4B-D). Substitution of E133 for Leu or Met is tolerated, probably because the extra methylene spacer in its side chain relative to Val eliminates steric clash of the branched portion of their side chains with Asn 127 and 135 (Figure 4E and F). Although normally destabilizing, substitution of E133 for Gly is tolerated, likely because it possesses no side chain with which to clash and the flexibility introduced by the Gly is offset by the preserved and extensive H-bonding network of the electrostatic loop and helix dipole capping by the zinc ion. Together, these data show an association between mutations at E133 that produce aggregation and the effects of the specific substitution on critical van der Wäals interactions and, or, hydrogen bond networks.

## Conclusions

In the present study, we investigated how mutations in SOD1 that cause the protein to aggregate may destabilize native structure. SOD1 is a highly conserved protein and disease mutations are often found in positions that are highly conserved (Wang *et al.* 2006), begging the question of whether any amino acid substitution at these positions could cause the protein to aggregate. Similar to the disease-associated mutation E40G (ALS), mutations E40D and E40V induced aggregation. Disease-causing mutations at Glu 133, in-frame deletion or mutation to V, induced aggregation as expected; whereas three non-conservative mutations (G, L, and M) were as well-tolerated as was a conservative E133D mutation. Analysis of the local structure around positions 40 and 133 suggested that mutations that are predicted to disturb van der Wäals contacts or produce steric strain are associated with a higher propensity to aggregate (Table 1).

A study of a set of ALS mutations that are predicted to have minimal effects on structural stability also suggested that relatively subtle changes in structure can cause disease (Byström *et al.* 2010). Missense substitutions at surface residues D76, N86, D90, D101, and N139 have been identified in ALS patients. A subset of the known substitutions at these residues, such as D76V, N86K, D90V, were predicted to introduce steric strain; whereas as others, such as D76Y, N86D, N86S, and D101N were predicted to diminish critical hydrogen bonds or salt bridges between residues in different elements of the protein (Byström *et al.* 2010). Two ALS mutations in D101 are particularly interesting as D101G was predicted to eliminate van der Wäals interactions whereas D101N was predicted to be sterically neutral and affect only electrostatic interactions (Byström *et al.* 2010). In a prior study that compared the aggregation propensity of D101G and D101N, we observed that D101G variant showed a more rapid rate of aggregation in cultured cell models (Ayers *et al.* 2014a). In the data we present here, we find that the E40G mutation, which is also predicted to eliminate critical van der Wäals interactions while also introducing flexibility in the peptide backbone was highly prone to form inclusions (see Table 1). Interestingly, to our knowledge the E40G mutation has been found only in one patient that was described as having a slowly progressing disease (Bertolin *et al.* 2014); whereas the D101G and D101N mutations are associated with rapidly progressing disease (Prudencio *et al.* 2009).

Although the nature of the toxic form of mutant SOD1 in fALS remains imprecisely defined, it is clear that one consequence of disease-causing mutations in SOD1 is to destabilize normal structure in a manner that facilitates aberrant homotypic self-assembly into higher order structures (Wang *et al.* 2009; Ivanova *et al.* 2014). Some pathogenic SOD1 variants, such as substitutions in the metal-binding loop elements [*e.g.* H80R, D124V (Seetharaman *et al.* 2009) and S134N (Elam *et al.* 2003)] cause reduced metal-binding (and as a consequence, reduced S-S oxidation) to promote monomerization (Arnesano *et al.* 2004; Doucette *et al.* 2004; Lindberg *et al.* 2004). For other variants, such as those studied here and by Byström *et al.* (2010), the impact of the specific substitution on protein structure appears to be more subtle and in some cases the effect is limited to disruption of local van der Wäals interactions or hydrogen bonds. Additionally, it has been suggested that mutations could induce structural changes that promote oligomerization in prelude to adopting more misfolded conformations and aggregation (Healy 2015). Evidence of oligomerized mutant SOD1 dimers has been observed in X-ray crystallography (Elam *et al.* 2003). A key observation in assessing these divergent potential mechanisms of mutant SOD1 aggregation is that regardless of the location of the mutation, the protein that appears to comprise such aggregates lack critical post-translational modifications, including incorporation of Cu and formation of intramolecular disulfide bonds (Jonsson *et al.* 2006a; Karch *et al.* 2009; Lelie *et al.* 2011). Given the importance of post-translational modification in the stability of SOD1, there could be examples of mutations that have little impact on SOD1 structurally and instead alter some key interactions with the copper chaperone for SOD1, leaving the protein deficient in Cu, less mature, and vulnerable to misfolding (Winkler *et al.* 2010).

In a prior study of 11 different ALS mutations, Bruns *et al.* found that mutant SOD1 exhibited delayed folding kinetics without necessarily abrogating folding into conformations closely resembling WT SOD1 (Bruns and Kopito 2007). Recently, we observed that newly made ALS-mutant SOD1 was rapidly captured into growing cytoplasmic inclusions (Ayers *et al.* 2017). Immature SOD1, lacking an intramolecular disulfide bond, shows greater structural disorder than mature protein with an intact disulfide bond (Furukawa *et al.* 2016). In the context of these observations, the seeming subtle effects of aggregation-inducing substitutions in SOD1 on local folding could be amplified early in maturation of the protein to favor off-pathway folding into aggregation prone conformations.

**Figure 5:**
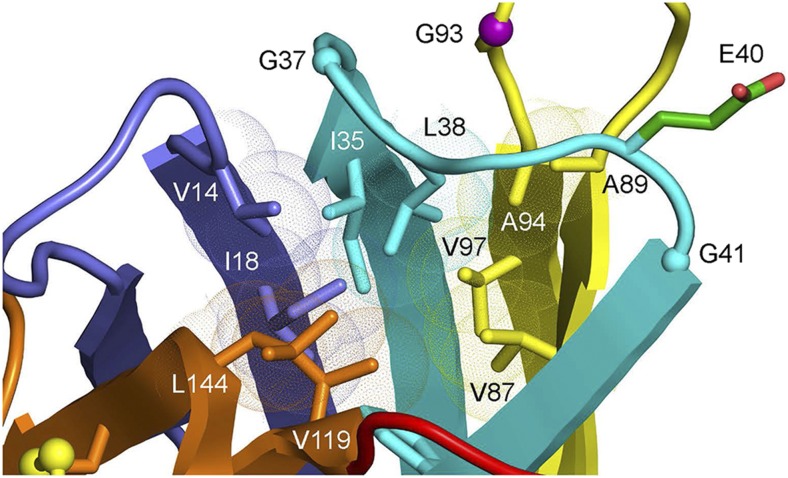
View of the local structure in the β-barrel plug of SOD1. Leu 38 forms the “plug” that seals the β-barrel structure. fALS mutations occur at multiple residues near L38, including L38 itself, G37, G93, G41, and V97 {http://alsod.iop.kcl.ac.uk}.

Recently, Ivanova and colleagues identified several short segments of SOD1 sequence possessing a high propensity to form amyloid fibrils (Ivanova *et al.* 2014). These segments are solvent inaccessible in the mature, homodimeric form of the enzyme. Two of the segments, ^14^VQGIINFE^21^ and ^30^KVWGSIKGL^38^, are involved in alignment of the β-barrel plug residue L38 and the apolar residues with which it interacts (V14, I18, I35, Figure 5). Nascent SOD1 proteins harboring destabilizing pathogenic substitutions in the β-barrel plug region *(e.g.* positions 38, 40, 93, and in the surrounding region; see Figure 5) are significantly destabilized relative to their wild type counterparts (Rodriguez *et al.* 2005), suggesting these segments may be more solvent-exposed and amenable to self-assembly in these variants. A third segment, ^147^GVIGIAQ^153^, is buried at the homodimer interface in the mature enzyme, but exposed to bulk solvent in newly translated, monomeric forms (Seetharaman *et al.* 2009). Our findings fit with a model in which aggregation-causing substitutions at positions 40 and 133 introduce steric clashes and, or, destabilize van der Wäals interactions; thereby slowing maturation of the protein and increasing the probability of aberrant self-assembly mediated by inherently amyloidogenic sequences in SOD1.
